# Urinary Exosomal miR-193a Can Be a Potential Biomarker for the Diagnosis of Primary Focal Segmental Glomerulosclerosis in Children

**DOI:** 10.1155/2017/7298160

**Published:** 2017-01-29

**Authors:** Zhibin Huang, Yong Zhang, Jianhua Zhou, Yu Zhang

**Affiliations:** Department of Pediatrics, Tongji Hospital, Tongji Medical College, Huazhong University of Science and Technology, Wuhan 430030, China

## Abstract

*Background*. Glomerular upregulation of miR-193a has been detected in primary focal segmental glomerulosclerosis (FSGS) but not in other glomerular diseases. We aimed to isolate exosomes from urine of children with primary FSGS and to assess the diagnostic potential of urinary exosomal miR-193a for primary FSGS.* Methods*. The first morning urine samples were collected from children with primary FSGS (*n* = 8) and minimal change disease (MCD, *n* = 5). Isolated urinary exosomes were confirmed by electron microscopy and Western blotting. Urinary exosomal microRNA was extracted, and the expression levels of exosomal miR-193a were quantified by real-time PCR. The diagnosis value of urinary exosomal miR-193a levels for primary FSGS was evaluated by ROC analysis.* Results*. The isolated vesicles were qualitatively compatible with exosomes. The levels of urinary exosomal miR-193a were significantly higher in children with primary FSGS than those in children with MCD. Moreover, the area under the ROC for the diagnosis of primary FSGS using urinary exosomal miR-193a was 0.85.* Conclusions*. A significant increase in the levels of urinary exosomal miR-193a in primary FSGS patients compared to those in MCD ones was observed. This study suggests that urinary exosomal miR-193a may be a new noninvasive biomarker for the diagnosis of primary FSGS.

## 1. Introduction

Focal segmental glomerulosclerosis (FSGS) and minimal change disease (MCD) are the two most common pathologic entities caused by podocytopathy. FSGS is also one of the most common primary glomerular diseases leading to end-stage renal disease in children [1]. Two-four out of every 100,000 children are diagnosed with nephrotic syndrome each year. FSGS accounts for 15–20% of these cases and is characterized by podocytic injury resulting in focal segmental obliteration of capillary lumina by the accumulated extracellular matrix. Unlike MCD patients, most patients with FSGS have poor therapeutic response to corticosteroid use, although other immunosuppressive therapies are available.

Currently, renal biopsy is still the gold standard for the diagnosis and the classification of FSGS [2]. However, abnormal glomerular findings might be missed due to sampling inadequacy, and it might be difficult to distinguish early FSGS from MCD. In addition, this procedure is invasive in nature with potential complications, which can be a major concern for the children and their parents. Finally, monitoring the clinical course with repeated renal biopsy is difficult during practice. Hence, reliable noninvasive biomarkers for accurate diagnosis are urgently needed in children with FSGS. Several reports suggested that urine alpha 2 macroglobulin levels can differentiate children with FSGS from those with MCD, but ethnic differences preclude making any definite conclusion.

Exosomes are small (30–100 nm) vesicles released after the fusion of intracellular multivesicular bodies with cell membrane. They have been found in body fluids including urine, plasma, synovial fluid and are initially thought to be excreted cellular wastes during reticulocyte maturation. With the increased knowledge of microvesicles, researchers found that exosomes contain nucleic acid and proteins; they not only play a role in clearance of cellular wastes but also participate in intercellular communication [3]. In addition, specific microRNAs (miRNAs) are found to be enriched in exosomes in a cell-activation-dependent fashion. These findings lead to the hypothesis that exosomal miRNAs can be used as a potential diagnostic tool for different diseases. Several reports have already identified that certain exosomal miRNAs can serve as useful biomarkers [4–7]. Furthermore, Gebeshuber et al. found that transgenic expression of miR-193a in mice rapidly induces FSGS with extensive podocyte foot process effacement, and miR-193a levels were also upregulated in isolated glomeruli from adult patients with primary FSGS [8]. Subsequently, Kietzmann et al. reported that miR-193a is involved in the molecular switch between phenotypes of parietal epithelia and those of podocytes cells [9]. In addition, plasma miR-193a levels are also found to be higher in patients with FSGS than those in healthy controls [10]. None of the existing reports utilized urine biomarkers for the diagnosis of FSGS in children. Consequently, we assessed the diagnostic value of urine exosomal miR-193a for primary FSGS in children.

## 2. Materials and Methods

### 2.1. Participants and Sample Collection

The study was approved by the Ethical Committee of Tongji Hospital, and written informed consent was obtained from all patients. All urine samples were obtained from children with nephrotic syndrome treated with steroid at the department of pediatric nephrology of Tongji Hospital between January 2015 and January 2016. To minimize the impact of urine concentration on urinary exosomal miR-193a levels, we collected the first morning urine for analysis. In advance, we set exclusion criteria to reduce the effects of systemic status on the miRNAs expression. The exclusion criteria included genetic testing-proven genetic FSGS, secondary FSGS, urinary tract infection, positive family history of kidney diseases, congenital anomalies of the kidney and urinary tract, nephrotic syndrome of secondary origin, diabetes, hypertension, nonnephrotic proteinuria, estimated glomerular filtration rate (eGFR) < 90 mL/min/1.73 m^2^ (calculated using the Schwartz formula), and treatment with immunosuppressant. Simultaneously, clinical data including age, gender, body height, body weight, 24-hour proteinuria amount, serum albumin level, and the type and dose of steroid were recorded.

### 2.2. Exosome Isolation and Exosomal RNAs Extraction

Early morning urine samples in the amount of 10 mL were collected from each patient in urine collection tubes and were processed within 2 h. The exosomes were isolated from urine using ExoQuick exosome precipitation (System Biosciences, Mountain View, CA, USA). Briefly, urine was centrifuged at 3,000*g* for 15 min to remove cells and debris. Supernatant was transferred to sterile vessels, and appropriate volume of ExoQuick-TC Exosome Precipitation Solution was added. The mixture was then refrigerated overnight (for at least 12 h), followed by another centrifuge at 1,500*g* for 30 min at 4°C. We aspirated the supernatant, spun down the residual ExoQuick-TC solution by centrifugation at 1,500*g* for 5 min, and removed all traces of fluid. Finally, 200 *μ*L of resuspended exosomal pellets was subjected for RNA analysis, using a Total Exosome RNA and Protein Isolation Kit (Invitrogen, Carlsbad, CA, USA), according to the manufacturer's protocol. The RNA concentration and purity were assessed by measuring the relative absorbance ratio at 260/280 using the Nanodrop 2000 (Thermo, Wilmington, DE, USA).

### 2.3. Exosome Identification by Electron Microscopy and Western Blotting

Exosome pellets were fixed with 100 *μ*L glutaraldehyde and mounted onto Formvar/carbon-coated EM grids. The grids were then transferred and stained with 0.75% uranyl formate for 30 s and then dried with filter paper. Samples were imaged on the transmission electron microscopy (Tecnai 20, FEI, Hillsboro, OR, USA), with sizes measured by image (NIH).

Isolated exosomes were lysed in RIPA buffer and centrifuged at 200,000*g* for 10 min. Supernatants were collected and heated at 100°C for 5 min and loaded onto 12% SDS-polyacrylamide gels. Protein was electrophoretically transferred to PVDF membranes, blocked, and then incubated overnight with primary antibodies against CD9 (dilution 1 : 1000, Abcam, Cambridge, MA, USA) and HSP70 (dilution 1 : 1000, Abcam, Cambridge, MA, USA), followed by HRP-conjugated secondary antibody (Santa Cruz Biotechnology, Delaware, CA, USA). The blots were analyzed by densitometric scanning using the WCIF ImageJ software.

### 2.4. Quantitative Real-Time Polymerase Chain Reaction (qRT-PCR) Quantification of miRNAs

miRNA was reverse-transcribed to cDNA using a miRNA cDNA Synthesis Kit (ABM Inc., Richmond, BC, Canada), and the qPCR reaction was performed using EvaGreen qPCR Master mix (ABM Inc., Richmond, BC, Canada). The primer mix for RNU6 and has-miR-193a-5p were obtained from Genecopoeia lnc. (Rockville, MD, USA). The PCR conditions were one cycle at 95°C for 10 min, followed by 40 cycles at 95°C for 10 s, 63°C for 15 s, and finally 72°C for 32 s. Exosomal miR-193a levels were normalized to RNU6 and were described as the ratios to the scramble control.

### 2.5. Statistical Analysis

Statistical analysis was performed by GraphPad Prism version 6 (GraphPad Software Inc., La Jolla, CA, USA). Raw threshold cycles (Ct) values were imported from ABI7500 SDS software (Applied Biosystems, Foster City, CA, USA), and relative expression levels for each miRNA were calculated using the comparative Ct method. Mann–Whitney* U*-test was used to compare gene expression levels between different groups, and ROC curve was utilized to assess the diagnostic performance of urinary exosomal miR-193a. *P* < 0.05 was considered statistically significant. All of the results are presented in means ± standard deviation.

## 3. Results

### 3.1. Morphological and Biochemical Characterization of Exosomes from Urine Samples

**Figure 1:**
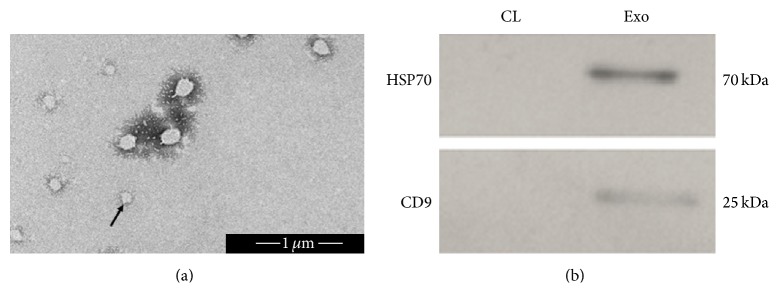
Identification of the extracellular vesicles. (a) Morphological characteristics of extracellular vesicles isolated from urine under transmission electron microscopy (scale bars: 1 *μ*m). Most vesicles show the characteristic exosomal spherical structure and size (diameter < 150 nm). (b) The detection of exosomal markers (Hsp70, CD9) by Western blotting. Data are representative images from the same FSGS patient. CL, urinary cellular lysates; Exo, urinary exosomes.

In this study, we adopted an exosome one-step precipitation protocol as a standard procedure for isolating exosomes from urine. The obtained extracellular vesicles were subjected to electron microscopy examination and Western blotting to confirm their nature. The vesicles (Figure 1(a)) we retrieved were spherical structures with diameters between 50 and 150 nm. To further verify whether these vesicles were exosomes, we assayed the molecular markers of exosomes using Western blotting. Two specific bands, CD9 and HSP70, were detected at 25 and 70 kD (Figure 1(b)). Therefore, the isolated extracellular vesicles fitted the consensus profile of exosomes with acceptable qualities and were suitable for subsequent experiments [11].

### 3.2. Determination of Exosomal miR-193a Expressions in Children with Primary FSGS and with MCD

**Figure 2:**
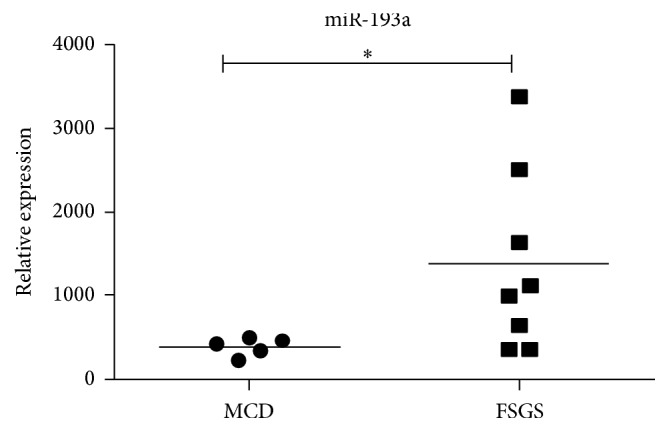
Comparison of urinary exosomal miR-193a levels between children with primary FSGS and those with MCD as a control. The level of urinary exosomal miR-193a was significantly higher in the primary FSGS group compared to those in the MCD group (1376 ± 382.3 versus 386.3 ± 47.66). Quantitative data represents the relative miR-193a levels to those of RNU6. ^*∗*^*P* < 0.05.

Eight biopsy-proven primary FSGS patients and five clinical definite MCD patients (MCD patients do not usually have a biopsy as most are steroid sensitive) satisfied the inclusion and exclusion criteria of this study between January 2015 and January 2016. Upregulation of miR-193a was found in glomeruli of children with FSGS compared to those from normal kidneys or from patients with other glomerular diseases (IgA nephropathy, membranous nephropathy, and MCD) [8]. We further collected the first morning urine samples from 8 children with FSGS and compared their findings to those from MCD patients. Simultaneously, we evaluated the clinical characteristics including age, gender, serum albumin level, 24-hour proteinuria amount, and the dose of steroid (Table 1). There were no significant differences in gender ratio, serum albumin level, proteinuria amount, or the dose of steroid between both groups. However, significant difference in age was observed (*P* < 0.05). The expressions of miR-193a from urinary exosomes were significantly higher in the primary FSGS group than those in the MCD group (*P* < 0.05) (Figure 2). This finding indicated that urinary exosome appeared to be a potential source for analyzing miR-193a levels for the differentiation between FSGS and MCD in children.

### 3.3. Receiver-Operating Curve (ROC) Analysis for the Diagnostic Values of Exosomal miR-193a for Primary FSGS

To evaluate the diagnostic values of urinary exosomal miR-193a for primary FSGS, ROC curves were generated to discriminate primary FSGS from MCD in children. We found an area under the ROC curve (AUC) of 0.85 (95% confidence interval [CI] 0.63–1.07) (Table 2). A ROC analysis identified an optimal threshold of urinary exosomal miR-193a for the diagnosis of FSGS at 530, with a high sensitivity of 75% and a high specificity of 80%. This finding indicated that urinary exosomal miR-193a may be a good index for the differentiation between primary FSGS and MCD in children.

## 4. Discussion

The pathogenesis of FSGS is characterized by podocyte injury. Podocytes are highly specialized epithelial cells interconnected by slit diaphragms, covering the outer layer of the glomerular basement membrane. Podocytes play a crucial role in stabilizing glomerular architecture and function, and exosomes derived from podocytes have been detected in urine. This suggests that urine exosomes can be an excellent source of biomarkers for evaluating renal pathology [12–15]. Thus we hypothesized that extracellular nucleic acids from urinary exosomes may function as important biomarkers for podocytopathy, and we derived comparable results.

MiRNAs are small (18–22 nucleotides), noncoding RNA molecules that modulate differentiation, growth, apoptosis, and proliferation of cells by negatively regulating gene expression posttranscriptionally. The dysregulation of miRNAs is associated with various diseases, including renal dysfunction [16–19]. Previous reports have demonstrated that miR-193a is directly implicated in the pathogenesis of FSGS [8]. Consistent with the Gebeshuber et al. study in renal tissues [8], we found that the level of urinary exosomal miR-193a was significantly higher in children with primary FSGS than that in children with another podocytopathy, MCD. The result of our ROC analysis (an AUC of 0.85) further lends support to the credibility of this association. Judging from our finding, testing the urinary exosomal miR-193a levels for the early diagnosis of primary FSGS might provide valuable and critical information. Interestingly, it has been reported that the target genes of miR-193a (such as Mcl-1) participate in the regulation of cellular apoptosis and autophagy [20–25]. Kawakami and colleagues' study also suggested that the failure of autophagy might be a pivotal mechanism in the pathogenesis of FSGS [26]. These findings triggered the hypothesis that miR-193a may drive the progression of FSGS by regulating autophagy in podocytes. Further studies are needed to clarify this point.

The key to identifying reliable exosomal biomarkers lies in the optimal exosome isolation process. In this study, we established a reliable and convenient protocol for isolating exosomes from the patients' urine using ExoQuick-TC kit. Although there are already several methods for isolating urinary exosomes available, the most commonly used one is based on ultracentrifugation [27]. However, ultracentrifugation has many shortcomings, including requiring expensive equipment, the lack of standardized parameters, and its time-consuming nature. In addition, most studies clearly indicate the possibility of failure to recover exosomal RNAs and proteins through ultracentrifugation [28, 29]. These defects limit its applicability during clinical practice. On the other hand, the use of polymer solutions to isolate exosomes is introduced at a later time. Several commercial products have been developed to isolate exosomes through polymer-based precipitation, and the most commonly used one is ExoQuick-TC from System Biosciences. Alvarez compared this urinary exosome-isolating method to the others and found that ExoQuick is the simplest, fastest, and most effective alternative to ultracentrifugation-based protocols, if the study goal is to collect RNA for profiling [30]. Therefore, the protocol for exosome isolation and identification in this study may exemplify the use of miRNA detection from urinary exosomes for patients with renal diseases.

However, there are some limitations to this study. Firstly, the number of patients was relatively low, and further studies are necessary to validate the results. Secondly, there was significant difference in age between FSGS and MCD groups because this study design was cross-sectional and the limited number of participants did not cover wide range of age. Thirdly, the relationship between urinary exosomal miR-193a and renal tissue miR-193a was not investigated. Fourthly, long-term follow-up data for children with FSGS and for those with other podocytopathy (such as membranous nephropathy) was unavailable.

In conclusion, we obtained two important findings from this pilot study, different from previous reports of children with FSGS. First, this is a preliminary report focusing on the diagnostic value of urinary miR-193a for children with primary FSGS. Second, we demonstrated the potential of using urinary exosomal miRNAs as a novel approach for the diagnosis of renal diseases, particularly FSGS.

## Figures and Tables

**Table 1:** Clinical characteristics of the subjects (*n *= 13).

Patient	Age	Gender	24-hour proteinuria	Clinical diagnosis	Pathologic diagnosis	Treatment
(1)	3 y 5 m	F	2840 mg	SSNS	NA	PED Oral 2 mg/kg/d
(2)	3 y 5 m	M	9643 mg	SSNS	NA	PED Oral 2 mg/kg/d
(3)	2 y 2 m	F	1184 mg	SSNS	NA	PED Oral 2 mg/kg/d
(4)	1 y 10 m	F	1064 mg	SSNS	NA	PED Oral 2 mg/kg/d
(5)	3 y	M	1476 mg	SSNS	NA	PED Oral 2 mg/kg/d
(6)	5 y	F	2121 mg	SRNS	FSGS (NOS)	PED Oral 2 mg/kg/d
(7)	12 y 11 m	M	1477 mg	SRNS	FSGS (NOS)	PED Oral 2 mg/kg/d
(8)	6 y 8 m	M	6000 mg	SRNS	FSGS (NOS)	PED Oral 2 mg/kg/d
(9)	9 y 2 m	M	3522 mg	SRNS	FSGS (NOS)	PED Oral 2 mg/kg/d
(10)	9 y 4 m	M	2430 mg	SRNS	FSGS (NOS)	PED Oral 2 mg/kg/d
(11)	5 y	M	1438 mg	SRNS	FSGS (NOS)	PED Oral 2 mg/kg/d
(12)	11 y 2 m	M	13116 mg	SRNS	FSGS (collapsing)	PED Oral 2 mg/kg/d
(13)	5 y	F	1894 mg	SRNS	FSGS (NOS)	PED Oral 2 mg/kg/d

F, female; M, male; SSNS, steroid-sensitive nephrotic syndrome; SRNS, steroid-resistant nephrotic syndrome; NOS, not otherwise specified variant; PED Oral, prednisone oral; NA, not available.

**Table 2:** Levels of miR-193a in urinary exosomes in children with FSGS and with MCD.

	FSGS (*n* = 8)	MCD (*n* = 5)	Fold change	AUC	*P* value
miR-193a	1376 ± 382.3	386.3 ± 47.66	3.56	0.85	0.04

Values are expressed as means ± standard error (SE). AUC, area under the receiver-operating curve.
